# Outcome of Methanol Toxicity Outbreak In Saudi Arabia: Case Series Study

**DOI:** 10.7759/cureus.41108

**Published:** 2023-06-28

**Authors:** Abdulghani O Kabli, Abdullah M Felemban, Alanoud K Nasri, Faisal A Aljedani, Ahmed M Bedairi, Mohammed M Alghamdi, Abdullah S Alghamdi, Saeed Y Ogran

**Affiliations:** 1 Intensive Care Unit, King Fahad General Hospital, Jeddah, SAU; 2 Internal Medicine, Dr. Soliman Fakeeh Hospital (DSFH), Jeddah, SAU; 3 Intensive Care Unit, King Abdulaziz Hospital, Jeddah, SAU; 4 Intensive Care Unit, East Jeddah Hospital, Jeddah, SAU

**Keywords:** metabolic acidosis, fomepizole, vision loss, toxicity, methanol

## Abstract

Introduction

Methanol poisoning is an acute medical emergency. If not recognized in time and treated properly, it can lead to a considerable magnitude of morbidity as well as mortality. This article aims to report cases of methanol toxicity, focusing on clinical presentation, management, and outcomes.

Method

Nine ICU-admitted patients with confirmed positive serum methanol levels were analyzed in a case series at King Fahad General Hospital in Jeddah, Saudi Arabia, between November 2022 and January 2023.

Results

Among the nine patients admitted to the ICU due to methanol poisoning, the majority were middle-aged males, with two females included. Gastrointestinal symptoms were seen in two-thirds of patients, while three patients required immediate mechanical ventilation due to a low Glasgow Coma Scale. Severe metabolic acidosis was observed in most cases. All patients received sodium bicarbonate and hemodialysis, with six patients also receiving fomepizole. However, two patients in the study with low initial low Glasgow Coma Scale (GCS), severe metabolic acidosis, and diffuse brain edema, did not survive. One patient reported acute complete vision loss.

Conclusion

This case series highlights the importance of promptly recognizing and managing methanol toxicity in ICU settings. The clinical presentation of methanol toxicity can be challenging, and early diagnosis and treatment are crucial to prevent irreversible damage.

## Introduction

Methanol is a clear and colorless liquid commonly used in industry as an antifreeze and wiper fluid. It is also found in colognes and perfumes [1]. Methanol is cheap and easy to obtain, making it a common ingredient in the production of illegal alcoholic beverages. In Saudi Arabia, where the distribution and sale of alcohol are legally prohibited, methanol poisoning is largely due to the consumption of underground handmade alcoholic beverages [2].

Methanol toxicity is a serious medical emergency that can cause severe central nervous system depression, metabolic acidosis, and blindness, and can even lead to death if not treated promptly [3]. Its Toxicity results from its metabolism by alcohol dehydrogenase (ADH) to formic acid, which accumulates and results in metabolic acidosis and organ injuries. Small ingested amounts of as little as 10 mL of pure methanol may be sufficient to cause life-threatening toxicity and permanent blindness [4].

Methanol toxicity can present a wide range of symptoms that may overlap with other toxicities or diseases, making it challenging to diagnose, especially in cases where medical history is not immediately available [5]. Therefore, a high degree of suspicion and early diagnosis is critical to preventing irreversible damage caused by formic acid. Prompt knowledge and early treatment are essential since the degree of damage is time-sensitive.

Methanol toxicity is a global public health problem that affects both industrialized and developing countries. In Saudi Arabia, methanol toxicity cases have been reported in various regions, and methanol toxicity outbreaks have also been reported [5, 6]. Therefore, the objective of this case series article is to report on cases of methanol toxicity encountered in our hospital and to highlight the clinical presentation, management, and outcomes of patients with methanol poisoning.

## Materials and methods

This is a case series of nine consecutive intensive care unit (ICU) admitted patients with methanol poisoning seen between November 2022 and January 2023 at King Fahad General Hospital, public tertiary and central hospital of Jeddah City, Western Region, Saudi Arabia. Clinical data was obtained from patients' files, including demographic and clinical information, laboratory investigations, treatment modalities, and ICU outcomes. Methanol poisoning diagnosis was confirmed by typical symptoms, signs, and positive methanol consumption samples with excluding all others. Data retrieval was approved by the research ethical committee.

## Results

This case series included a total of nine patients who were admitted to the ICU and their details were tabulated in. Of the nine patients, two were female; the majority were middle-aged, ranging from 30 to 52 years old. The route of toxicity was through drinking for all of them, with gastrointestinal symptoms observed in two-thirds of them. One patient reported acute complete vision loss, while a low initial Glasgow Coma Scale was observed in three patients who required immediate mechanical ventilation (Table 1).

**Table 1:** Clinical Parameters Of The Series Upon Presentation. LOC: Level Of Consciousness; GCS: Glasgow Coma Scale; GITs: gastrointestinal tract Symptoms (Nausea, Vomiting and abdominal pain); SBP/DBP: Systolic Blood Pressure/Diastolic Blood Pressure.

	Clinical Parameters upon presentation								
	Case 1	Case 2	Case 3	Case 4	Case 5	Case 6	Case 7	Case 8	Case 9
Age (Years)/sex	52/Male	40/Male	39/Female	39/Female	41/Male	33/Male	30/Male	32/Male	44/Male
Presenting symptoms	GITs, Vision changes	GITs, Vision changes	GITs, decrease LOC	Chest discomfort, vision changes	GITs, complete vision loss	GITs, Vision changes	Confusion and drowsiness	Confusion drowsiness, vision changes	GITs, Vision changes
Heart rate (bpm)	120	72	110	113	97	82	110	90	80
SBP/DBP (mmHg)	195/105	175/95	105/89	115/70	167/100	125/75	140/80	145/78	135/85
Rout of exposure	Drinking	Drinking	Drinking	Drinking	Drinking	Drinking	Drinking	Drinking	Drinking
GCS	11	15	5	15	15	15	5	13	15

All patients had confirmed positive serum methanol levels, with a mean of 142 mg/dL (range 13-155 mg/dL), and most had severe metabolic acidosis. Only two patients had a high renal profile, and one had mildly elevated liver enzymes (Table 2).

**Table 2:** Biological And Laboratory Markers Of The Series. HCO3: Bicarbonate, Pco2: partial pressure of carbon dioxide, BUN: Blood urea nitrogen, AST/ALT: Aspartate aminotransferase/alanine aminotransferase.

	Biological and laboratory markers								
	Case 1	Case 2	Case 3	Case 4	Case 5	Case 6	Case 7	Case 8	Case 9
Serum methanol Level (mg/dL)	127	23	155	13	65	22	92	30	29
Arterial PH/Anion Gap	7.02/43.5	7.05/28	6.7/30	7.18/27.5	7.11/26	7.15/23	6.99/30	6.97/27	7.13/28
Arterial HCO3 (mEq/L)	4.5	7	5.2	9.9	8.1	10.7	3.9	2.1	7.1
Arterial Pco2 (mmHg)	25	16	41	21.4	10.9	28.2		19	15
Glucose (mg/dL)	400	269	238	115	220	97	92	150	120
Creatinine/BUN (mg/dL)	1.1/11	1.8/10	1.9/18	0.85/15.6	1.1/7	0.9/15	0.9/16	1.2/10	1.07/19
AST/ALT (U/L)	27/28	18/	98/96	39/25	44/28	19/14	67/60	47/30	42/41

Regarding management, all patients received hemodialysis and sodium bicarbonate, and six patients received fomepizole (Table 3). Unfortunately, two patients died, primarily due to low initial low Glasgow Coma Scale (GCS), severe metabolic acidosis, and diffuse brain edema (Table 4).

**Table 3:** Treatments Modalities

	Treatments								
	Case 1	Case 2	Case 3	Case 4	Case 5	Case 6	Case 7	Case 8	Case 9
Mechanical ventilation	Yes	No	Yes	No	Yes	No	Yes	Yes	No
Inotropes	Yes	No	No	Yes	Yes	No	Yes	Yes	Yes
Fomepizole	No	No	No	No	Yes	Yes	Yes	Yes	Yes
Hemodialysis started within	12H	15H	8H	19H	6H	4H	6H	5H	6H
Sodium bicarb	Yes	Yes	Yes	Yes	Yes	Yes	Yes	Yes	Yes
Steroid	Yes	Yes	No	Yes	Yes	No	Yes	Yes	Yes
Folic acid	Yes	No	Yes	No	No	No	Yes	Yes	No

**Table 4:** Outcome And Complications.

	Outcome and complication								
	Case 1	Case 2	Case 3	Case 4	Case 5	Case 6	Case 7	Case 8	Case 9
Brain involvement on images	Diffuse edema	No	Basal Ganglia, Diffuse edema and herniation	No	Basal Ganglia involved	No	Basal Ganglia involved	Basal Ganglia involved	Basal Ganglia involved
Outcome	Death	Improved	Death	Improved	Blindness	Improved	Improved	Blindness	Improved
Length of Hospital stay	7 Days	16 Days	3 Days	7 Days	18 Days	2 Days	16 Days	36 Days	5 Days

## Discussion

Methanol poisoning is an acute medical emergency. If not recognized in time and treated properly, it can lead to a considerable magnitude of morbidity as well as mortality. This article aims to report cases of methanol toxicity, focusing on clinical presentation, management, and outcomes of patients with methanol poisoning.

Based on our case series of nine patients with methanol toxicity, it was observed that the majority of the patients were male, with only 20% being female. Nevertheless, all of the patients are Middle Ages. This finding is consistent with reports from other studies conducted in Saudi Arabia, as well as in other Arabic and Asian countries [3, 6-11]. It is possible that social and religious norms play a role in limiting female involvement in methanol poisoning.

Common symptoms of methanol consumption include central nervous system (CNS) depression, gastrointestinal symptoms, metabolic acidosis, and visual disturbances. Symptoms can appear up to 24 hours after ingestion, and a longer half-life may occur with higher concentrations and co-ingestion with ethanol [12], All of our nine patients had severe metabolic acidosis, the most common disturbance seen in methanol intoxication due to formic acid accumulation. Visual disturbances, the only specific symptom of methanol poisoning, were present in approximately two-thirds of patients [13]. Three patients had very disturbed consciousness levels with a Glasgow Coma Scale ranging from 4-11, which could be confusing for emergency physicians. Similar findings of rapid deterioration in consciousness have been reported in other studies, including a case report from Saudi Arabia, even brain dead after a toxic dose has been as well [10, 14-15].

Methanol's toxicity comes from its metabolites, particularly the accumulation of formic acid, which is responsible for the pathological changes in methanol poisoning, The complicated association between serum methanol concentration and its clinical effects makes interpretation challenging and most of the published case series lack the methanol serum level [6]. The fatal amount of methanol is approximately 1-2 mL/kg. However, even a small amount of 0.1 mL/kg (6-10 mL in adults) can result in permanent blindness and death [16]. Half of the patients in our series exhibited severe methanol poisoning with levels exceeding 50mg/dL, according to the literature-based classification that defines mild poisoning as less than 10mg/dL and moderate poisoning as between 10-50mg/dL [17, 18]. All patients in our case series presented with severe metabolic acidosis, with the patient who died having the most severe metabolic acidosis and high renal and liver profile, which have been associated with mortality in other case series [19].

Prompt recognition, treatment, and occupational safety are critical in managing methanol toxicity, the main objective of acute methanol intoxication treatment is to eliminate methanol and prevent complications [20]. Fomepizole is a potent inhibitor of alcohol dehydrogenase activity on methanol, which can prevent metabolic acidosis and reduce the incidence of ocular toxicity. Early fomepizole administration protected against damage and improved outcomes in some patients. However, some patients in our series did not receive it due to late presentation, diagnosis, or availability. This is consistent with other local series [19, 6].

Folic acid and folinic acid can convert formic acid into non-toxic byproducts. Folate is relatively safe and can be considered an adjunctive treatment for methanol poisoning, particularly for patients with metabolic acidosis with an anion gap [21]. Intermittent hemodialysis was recognized as the modality of choice, while continuous modalities were considered acceptable alternatives based on the international extracorporeal treatment in the poisoning work group. In our series, all patients presented at least one of the international extracorporeal treatments in poisoning work group criteria and were therefore dialyzed [22].

Metabolic acidosis has been identified as a stronger predictor of morbidity and mortality in methanol poisoning, compared to serum methanol concentration [19]. Methanol poisoning often leads to bilateral putamen necrosis and can produce diffuse bilateral white matter hypodensities. Two patients in our series with initial low GCS, severe metabolic acidosis, and diffuse cerebral edema died from methanol poisoning. These are poor prognostic factors that require prompt attention and early intervention [23, 6, 19, 10, 11].

## Conclusions

This case series emphasizes the significance of early diagnosis and treatment of methanol toxicity due to its challenging clinical presentation. The prompt recognition and management of methanol toxicity in ICU settings are critical in preventing irreversible damage as it is a potentially fatal public health issue. To address this problem, public education and legislative control should be emphasized, and medical authorities should set and include strict local protocols for early diagnosis and management.
